# Carryover of Aflatoxin From Dairy Feed to Milk: A Systematic Review (1 January 2004–31 December 2024)

**DOI:** 10.1111/jpn.70092

**Published:** 2026-07-19

**Authors:** Queenta Ngum Nji, Richard Temitope Fagbohun, Oluwafemi Ayodeji Adebo, Patrick Berka Njobeh

**Affiliations:** ^1^ Department of Biotechnology and Food Technology, Faculty of Science, Mycotoxin Research Group University of Johannesburg Johannesburg South Africa; ^2^ Department of Biotechnology and Food Technology, Centre for Innovative Food Research, Faculty of Science University of Johannesburg Johannesburg South Africa

**Keywords:** aflatoxin, carryover, dairy feed, milk

## Abstract

Aflatoxins, toxic metabolites produced by *Aspergillus* species, are frequently reported contaminants of dairy cattle feed worldwide, particularly in tropical and subtropical regions where climatic conditions favor fungal growth, and they can be transferred into milk and dairy products. Their presence poses major public health risks, including liver cancer and growth impairment, among others. Understanding the extent of carryover from feed to milk is critical for food safety and regulatory control. A systematic review was conducted to synthesize evidence on aflatoxin carryover in dairy systems over the past two decades. Literature searches were performed in PubMed, Scopus, Web of Science, and Embase. Studies investigating aflatoxin levels in dairy feed and milk, as well as carryover rates, were included. Data extraction focused on contamination levels, feed treatment efficiency and carryover rates of aflatoxins as well as mitigation measures. Twenty‐seven eligible studies were identified. Aflatoxin B_1_ concentrations in dairy feed ranged from undetectable to 598 µg/kg. In milk, AFM_1_ levels reached up to 22.2 µg/kg, with reported carryover rates as high as 15%. The findings highlight variability in contamination across regions and production systems. This review underscores the continuing risk of aflatoxin in dairy products, identifies knowledge gaps and emerging threats, and emphasizes the need for improved interventions to safeguard food safety and public health.

## 1 Introduction

Aflatoxins, a group of highly toxic and carcinogenic mycotoxins produced by certain species of *Aspergillus* fungi (primarily *A. flavus, A. parasiticus*, and *A. nomius*), are a significant concern in the dairy industry due to their potential to contaminate dairy feed, and subsequently, milk and its products (Gallo et al. 2024; Patel 2024). Aflatoxins are common contaminants of agricultural commodities and co‐products such as cereals, oilseeds, and others which are main dairy feed constituents and represent a major source of dietary energy and nutrients to animals (Yu and Pedroso 2023). Dairy cattle feed is either forage or concentrate. Feed is composed primarily of forage and concentrate, supplemented with minerals, vitamins and various additives (Kemboi et al. 2020). The forage portion can include grazed grasses or cultivated forage crops, hay, legumes, silage, and(or) crop residues. Concentrates include nutrient‐dense ingredients and compounds such as cereal grains, oilseeds, their co‐products as well as upcycled feedstuffs from other industries, and commercially compounded blends in various physical forms. Co‐products such as DDGS (distillers dried grains with soluble) can be a particularly concerning potential source of mycotoxins in animal feed because the mycotoxins become concentrated up to three times compared to the original raw grain during the ethanol production process (Murtaza et al. 2025). Forage and concentrates feed to dairy cows can be contaminated with aflatoxins, which can be transferred into milk (Changwa et al. 2018). Additionally, contamination of feeds with aflatoxins poses a significant performance challenge, limiting milk production capacity and feed efficiency by reducing feed palatability and nutritive value and redirecting nutrients to respond to toxin challenge (Dey et al. 2023; Gasco et al. 2023; Gross 2022; Smith 2020).

When dairy animals consume aflatoxin‐contaminated feed, particularly aflatoxin B_1_ (AFB_1_), the toxin is metabolized in the liver and biotransformed into the hydroxylated metabolite called aflatoxin M_1_ (AFM_1_) and subsequently excreted in milk (Malissiova et al. 2024). This entire process is known as carryover and involves a series of complex processes often influenced by multiple factors such as the type and level of aflatoxin contamination in the feed, physiological status and breed of dairy animals, the efficiency of hepatic metabolism, and the processing and handling of milk and milk products. Subject to feed contamination level, studies showed AFM1 carryover was typically 1%–2% for low‐yielding cows (< 30 L milk/day) and could reach up to 6% only in high‐yielding cows (> 30 L/day) Ferrari et al. (2023). Following the ingestion of AFB_1_, AFM_1_ levels in milk rise and then decline rapidly once exposure stops, becoming undetectable within 12–24 h after the last intake. AFM_1_ typically reappears only when the animal is re‐exposed, and may be detectable again about 3 days after consuming contaminated feed (Battacone et al. 2009; Torres et al. 2022; Soriano del Castillo 2007). It is worth noting that among the different types of aflatoxins, aflatoxin B_1_ (AFB_1_) is the most potent and prevalent contaminant in livestock feed. The International Agency of Research on Cancer reported the toxicity of AFM_1_ to be almost equivalent to its parent compound (AFB_1_), leading it to be classified as a group 1 carcinogen (IARC 2012). Therefore, the presence of AFM_1_ in milk and dairy products is of food safety concern due to its hepatotoxic, mutagenic, and carcinogenic properties, which pose serious risks to human health even at low concentrations.

The dairy industry is a significant sector of the global food system, providing essential nutrients and calories to millions of people worldwide (Reyna and Arteaga 2022). The dairy farming system is heavily dependent on rational feeding regimens where high nutrient intakes are needed to optimize milk production, with most of the dairy feeds principally compounded to meet these very high energy, protein, and trace nutrient requirements. As mentioned earlier, the primary components of these diets are often contaminated with very high levels of aflatoxins. This makes the dairy industry very vulnerable to the impact of aflatoxin contamination, which can result in significant human exposure as well as economic losses. Once present in milk, AFM_1_ can persist in processed dairy products such as cheese, yogurt, butter, and powdered milk, potentially increasing human exposure through widespread dairy consumption. AFM_1_ is thermally stable, giving rise to its high prevalence in processed milk and other dairy products, leaving significant uncertainty about the AFM_1_ levels after pasteurization (Daou et al. 2020; Rama et al. 2015). This has given rise to contrasting opinions necessitating further research on the effects of processing of aflatoxin‐contaminated milk for each and every dairy product. For instance, in cheese processing, cream separation affects AFM_1_ concentration in the product since 80% is partitioned in the skim milk and 30% is associated with the non‐fat milk solids (casein) (Kaniou‐Grigoriadou et al. 2005). Multiple studies have reported no effect of cheese ripening and storage time on AFM_1_ levels (Fernandes et al. 2012; Iha et al. 2013; Nilchian and Rahimi 2012). However, other investigations have contrary opinions on increased AFM_1_ levels (Hassan et al. 2018; Montagna et al. 2008) during ripening. Conflicting reports also exist as to the impact of processing on FM_1_ levels in yoghurt. While no change was reported by some researchers (Blanco et al. 1993; Stoloff 1980; Van Egmond 1983), others showed significant decreases in AFM_1_ levels in yoghurt compared with those initially added to milk (Govaris et al. 2002). In the European Union, AFs in food and feed are regulated under stringent maximum limits established by legislation (e.g., Commission Regulation (EC) No 1881/2006 for food and Directive 2002/32/EC for feed) to minimize consumer exposure and safeguard animal and human health. Numerous reports involving raw milk and various milk products showed that AFM_1_ levels in some processed products (specifically cheese) were higher than levels in the parent raw milk (Esam et al. 2022; Özturk Yilmaz and Altinci 2018; Younis et al. 2016). While others have argued that processing has no effect on levels of AFM_1_ in milk and milk products, Shabansalmani and Movassaghghazani (2023) used AFM_1_‐contaminated raw milk in yoghurt and cheese processing, and AFM_1_ was detected in all the tested dairy products.

Aflatoxin contamination of dairy feed is widespread and has been attributed to low levels of awareness on the one hand and food insecurity realities on the other. As long as these issues are not addressed, dairy animals will continue to be fed with aflatoxin‐contaminated feed which will negatively affect the dairy industry in terms of economic losses and health effects. While higher proportions of feed samples contaminated with AFB_1_ concentrations exceeding the EU regulatory limits were linked to preserved feeds of cereal origin and feeds fortified with concentrates, feed samples of dry pasture (leaves, stalks) presented low AFB_1_ levels (Omeiza et al. 2018). Hence, for an effective and sustainable aflatoxin regulation in the dairy sector, strict regulation of dairy feed should be the starting point. Given the essential role of milk in daily nutrition, particularly for infants and vulnerable populations, stringent regulatory limits have been established by global health authorities, including the European Food Safety Authority (EFSA), the U.S. Food and Drug Administration (FDA), and the Codex Alimentarius. However, in many developing countries, enforcement of these limits remains a challenge due to inadequate monitoring, regulatory gaps, and limited access to mycotoxin mitigation strategies. High incidences of AFM_1_ contamination of milk and milk products have been reported in developing countries due to their lower regulatory standards enforcement (Diogenes et al. 2023; Sirma et al. 2018).

Recently, an increased interest in the carryover of aflatoxins from dairy feed to milk and its products has been observed (Jonathan et al. 2024; Nji et al. 2025; Shabansalmani and Movassaghghazani 2023; Zentai et al. 2023). While several studies have been conducted to investigate this issue, outcome of these studies is often inconsistent and contradictory. The need for a comprehensive review of the literature to summarize the current state of knowledge on this topic cannot be over‐emphasized. This systematic review aims to provide a comprehensive analysis of aflatoxin carryover from dairy feed to milk, synthesizing evidence from recent studies to assess contamination levels, treatment and mitigation strategies over the past two decades. By critically evaluating existing literature, this review will highlight knowledge gaps, emerging risks, and potential interventions to reduce aflatoxin contamination in dairy supply chains, ensuring food safety and public health protection.

## 2 Materials and Methods

A systematic review was conducted following PRISMA (Page et al. 2021). This systematic review was conducted in accordance with a predefined protocol registered in the PROSPERO database (CRD420250653146), with the research question formulated using the PICO (Population, Intervention, Comparison, Outcome) framework.

### 2.1 PICO Element Description

*P Population/Problem*: Dairy animals (dairy cows) consuming aflatoxin‐contaminated feed; milk and dairy products consumed by humans.

*I (Intervention/Exposure)*: Ingestion of aflatoxin B_1_ (AFB_1_) through contaminated dairy feed and the subsequent transfer (carryover) of AFB_1_ to aflatoxin M_1_ (AFM_1_) in milk and dairy products.

*C (Comparator)*: Studies with different contamination levels in feed, in milk and diverse treatment methods.

*O (Outcome)*: Carryover rates of aflatoxin and efficacy of treatment methods.

### 2.2 Inclusion and Exclusion Criteria

#### 2.2.1 Inclusion Criteria

The inclusion criteria for the review focused on original research articles published between 1 January 2004 and 31 December 2024. This 20‐year period was selected to capture a comprehensive and methodologically comparable body of evidence that reflects both historical progression and contemporary advancements within the field. Research conducted from 2004 onward benefits from improved analytical technologies, enhanced surveillance and reporting systems, and more clearly defined regulatory and scientific frameworks, thereby ensuring the reliability and relevance of the included studies. The end date of December 2024 was chosen to incorporate the most up‐to‐date literature available at the time of the review, ensuring that the synthesis reflects current knowledge and emerging trends. Eligible studies specifically addressed aflatoxin contamination in dairy cattle feed, cow milk, and the carryover process from feed to milk. Only studies involving dairy systems with cow feed and milk data were considered.

#### 2.2.2 Exclusion Criteria

The exclusion criteria for the review were as follows:

i. studies not published in English;
ii. studies focusing on aflatoxins in non‐dairy food products;
iii. studies lacking quantitative data on aflatoxin levels in animal feed or cow milk;
iv. studies published outside the defined time frame (1 January, 2004 to 31 December, 2024);
v. non‐primary research articles, including reviews, conference abstracts, theses, and books.

### 2.3 Search Strategy

Our search strategy was based on e‐resources. Databases PubMed, Scopus, Web of Science, Embase and Food Science and Technology Abstracts (FSTA) were used. The review search was restricted to these major bibliographic databases due to their comprehensive indexing of peer‐reviewed and methodologically robust studies, as well as their reproducible search functions. Broader platforms such as Google Scholar and institutional repositories were not included because of inconsistent indexing, variable search algorithms, and challenges in ensuring reproducibility and quality assessment. Although this approach may introduce a degree of publication bias by potentially excluding relevant grey literature (e.g., theses or conference proceedings), this limitation was mitigated through reference list screening of included studies to identify additional sources. A comprehensive search strategy was developed using keywords and Medical Subject Headings (MeSH) terms related to ‘Aflatoxin carryover in dairy,’ ‘Aflatoxin in milk and dairy products,’ ‘Aflatoxin M_1_ contamination,’ ‘Mitigation of aflatoxins in dairy feed,’ and ‘Regulatory limits for aflatoxin in milk.’ Boolean operators (AND, OR) were utilized to refine the search. The search terms included Aflatoxin OR Aflatoxin M_1_ OR Aflatoxin B_1_ AND Carryover OR Transfer OR Remainder OR Surplus OR Continuation AND Milk OR Dairy products OR Dairy Feed AND Aflatoxin M_1_ contamination AND Mitigation of aflatoxins in dairy feed AND Regulatory limits for aflatoxin in milk AND Regulatory limits for Feed.

### 2.4 Checklist and Guidelines for the Included Articles

The ‘PRISMA 2020 statement: An updated guideline for reporting systematic reviews’ was used (Page et al. 2021) (supporting material section). To evaluate the risk of bias, the Joanna Briggs Institute (JBI) checklist with a minor modification was used with a total score of 7. The JBI checklist was classified into three categories, including high concentration (6 and 7 score), medium concentration (5 scores), and low concentration (< 4 scores) studies. The risk of bias assessment was evaluated to see if the included studies’ prevalence and concentration influenced the results.

### 2.5 Data Extraction

Two parallel and independent screenings were conducted on the initial title and abstract using Covidence software. Studies which were relevant to our studies, that is, those that reported aflatoxin levels in feed and milk between 1 January, 2004 and 31 December, 2024, were selected for included articles. Aflatoxin levels in feed and milk and other data were extracted and recorded from selected studies using Covidence. To show the aflatoxin levels in feed and milk, a forest plot was utilized, and source heterogeneity explored by subgroup analysis. This approach provided valuable insights into the potential heterogeneity of treatment effects and was used to help identify which specific characteristics may influence our outcomes. Finally, independent reviewers screened ‘titles and abstracts’ for relevance. Full texts of potentially eligible articles were assessed for inclusion. Discrepancies were resolved through a round table discussion with the rest of the authors. The final articles were then included in the quantitative and qualitative synthesis of this review.

## 3 Results

The systematic review employed a comprehensive literature search strategy across four major electronic databases to ensure exhaustive retrieval of relevant studies concerning the carryover of aflatoxins from dairy feed into milk. A total of 228 articles were identified, comprising 136 from PubMed, 50 from Web of Science, 25 from Embase, and 17 from Scopus. These databases were strategically selected to encompass multidisciplinary research spanning the medical, toxicological, veterinary, and agricultural sciences. According to PRISMA (https://app.covidence.org) documentation (Figure 1), no additional records were identified through grey literature searches or citation chaining.

**Figure 1:**
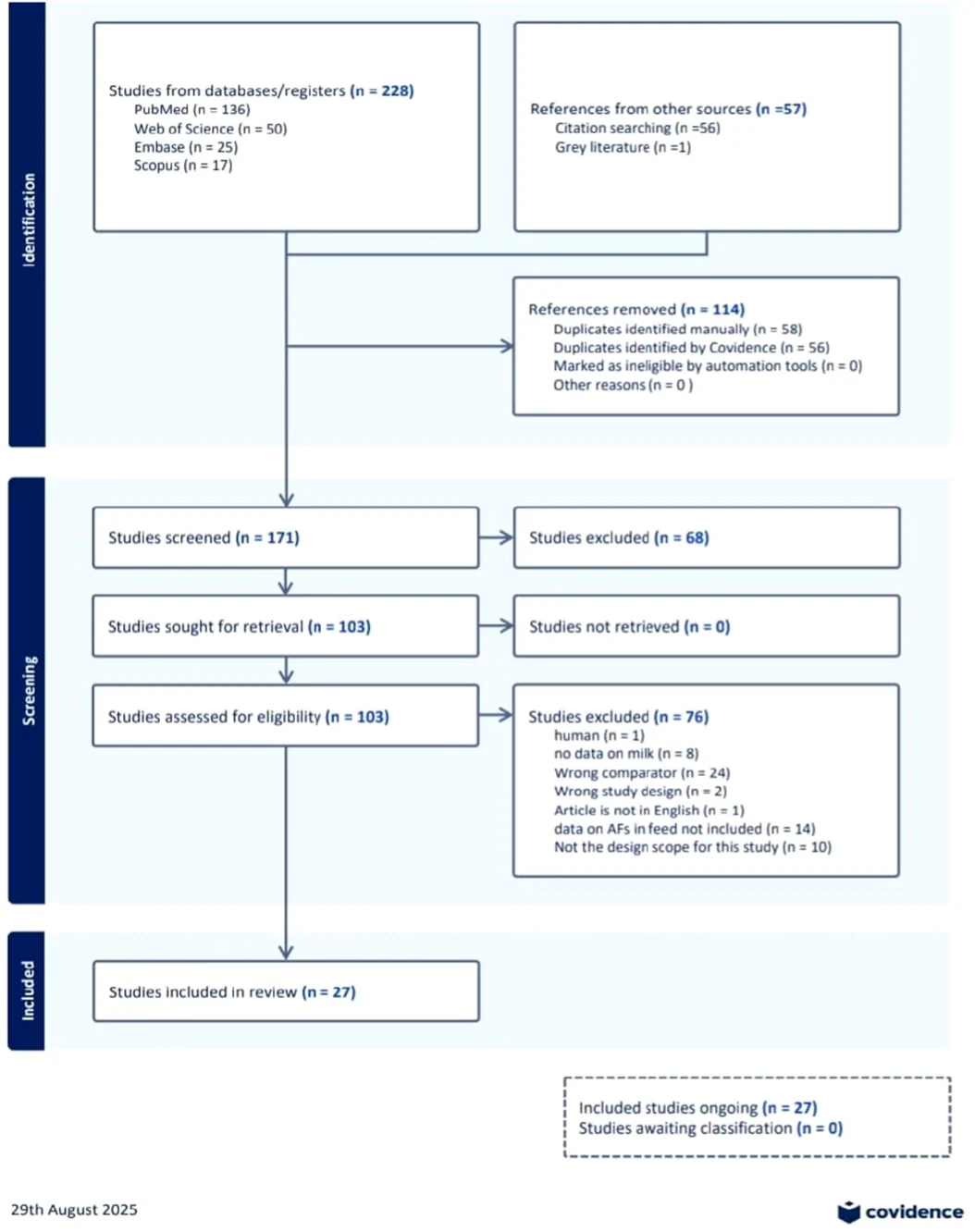
Flowchart of excluded and included studies based on the PRISMA Statement. [Color figure can be viewed at wileyonlinelibrary.com]

Following article identification, a multistage screening protocol was implemented. One hundred and seventy‐one articles proceeded to the initial screening phase, during which 56 duplicate entries arising from database overlap were detected and removed using Covidence, a software tool designed for systematic review management. This step ensured that only distinct records advanced to the eligibility assessment. Subsequently, 103 full‐text articles were retrieved and critically evaluated based on predefined inclusion and exclusion criteria. Of these, 76 articles were excluded for the following reasons: no data on either AFM_1_ in milk and (or) AFB_1_ in feed, wrong comparator, experimental design or scope not meeting defined criteria, and articles not in English.

Twenty‐seven studies were ultimately retained for synthesis following a final round of methodological appraisal or relevance verification. Table 1 presents all the relevant details extracted from these 27 articles according to this study design. These studies constitute the evidential foundation for evaluating the occurrence, concentration, and transfer rates of aflatoxins from dairy feed to milk over a two‐decade period, providing critical insights into exposure risks and control strategies as well as the knowledge gap in this regard.

**Table 1:** Select studies evaluating aflatoxin carryover from dairy cattle feed to milk over a 20‐year period.

S/N	Author(s)	Year	Country	Detection technique	AFB_1_ concentration (µg/kg)	AFM_1_ concentration (µg/kg)	Carryover rate (%)
1	Diaz et al.	2004	US	HPLC/ELISA	55	0.43–1.24	0.95–2.25
2	Masoero et al.	2007	Italy	HPLC	98.0–98.3	1.27–2.66	1.29–2.70
3	Oliveira et al.	2008	Brazil	HPLC	1.4–26.4	0.010–0.645	0.71–2.44
4	Kutz et al.	2009	US	HPLC	112	1.00–1.98	1.42
5	Polonelli et al.	2011	Italy	HPLC	10.8–144	0.095–0.18	0.13–0.88
6	Sumantri et al.	2012	Indonesia	ELISA	3.4–350	0.002–0.069	0.03–0.21
7	Britzi et al.	2013	Israel	LC‐MS/MS	84.1–89.8	0.02–0.256	2.1–15.2
8	Xiong et al.	2015	China	HPLC	20‐40	17.0–17.6	0.057–0.59
9	Gonçalves et al.	2017a	Brazil	HPLC	480	0.49–1.04	0.20–3.95
10	Sulzberger et al.	2017	US	ICP‐MS	100	0.35–22.82	0.5–1.01
11	Jiang et al.	2018	US	HPLC	16.0–28.5	0.40–0.75	0.93–1.65
12	Xiong et al.	2018	China	LC‐MS/MS	20	0.13–7.38	0.11–1.38
13	Pate et al.	2018	US	SNAP AFM_1_ kit	100	0.09–12.4	0.39–0.49
14	Guo et al.	2019	China	HPLC	63	0.48–0.67	0.76–1.06
15	Rodrigues et al.	2019	US	HPLC	101.4–107.6	8.1–22.2	1.0–2.7
16	Rodríguez‐Blanco et al.	2019	Spain	HPLC	0.05–6.45	0.009–1.36	0.6–6
17	Costamagna et al.	2019	Sweden	ELISA	1.9–8.1	0.003–0.064	0.05–5.93
18	Djekic et al.	2020	Serbia	ELISA	0.813–29.424	0.012–0.779	1.48–2.65
19	Intanoo et al.	2020	Thailand	ELISA	22.28	0.36–1.54	1.18–7.26
20	Cha et al.	2021	China	LC‐MS/MS	168	0.046–0.093	0.57–1.16
21	Bervis et al.	2021	Spain	HPLC	11.08–28.26	0.35–0.75	1.89–5.07
22	Walte et al.	2022	Germany	ELISA	50	0.018–0.070	1.22–4.43
23	Hassan H. Al‐Rubaye	2023	Iraq	ELISA	5.37–6.53	0.006–0.008	0.28–0.44
24	Kemboi et al.	2023	Kenya	UHPLC‐MS/MS	326–598	0.689–2.737	0.05–0.35
25	Jauković	2024	Serbia	ELISA	2.05–48.31	0.02–0.26	0.53–0.98
26	Costamagna et al.	2024	Sweden	ELISA	2.31	0.008–0.016	0.77–2.19
27	Borowsky et al.	2024	Brazil	UPLC	ND	0.03–032	NA

Abbreviations: ELISA, Enzyme‐Linked Immunosorbent Assay; HPLC, High‐performance liquid chromatography; ICP‐MS, Inductively Coupled Plasma Mass Spectrometry; LC‐MS/MS, Liquid Chromatography Tandem Mass Spectrometry; NA, Not applicable; ND, Not detected; UPLC, Ultra Performance Liquid Chromatography.

Table 1 provides a comprehensive overview of various studies investigating aflatoxin contamination in dairy feed and milk, specifically focusing on AFB_1_ and its transformation into AFM_1_ in milk. Spanning research conducted from 2004 to 2024, the table details the detection methods utilized, country where the study was conducted, detection techniques, contamination levels in both the feed and milk, and the rate at which aflatoxin was transferred into milk.

### 3.1 Distribution of Studies

Relatively few papers meeting study criteria were published prior to 2017. This could be attributed to the global awakening to the effects of climate change on toxigenic fungi and their overall effects on food systems. Furthermore, aflatoxin carryover from feed to milk studies are far from evenly distributed globally (Figure 2). While most of these studies have been reported in Europe (9), Africa had the least number of studies (1). Kemboi et al. (2023), evaluated the efficacy of bentonite in mitigating the effects of aflatoxins in two Kenyan cattle breeds. This is worrisome, as Africa is reportedly one of the regions most affected by the effects of aflatoxins (Nji et al. 2022). In a recent review on aflatoxin contamination of feed and dairy products, the rates and levels of contamination reported in African countries were very high and of particular concern (Nji et al. 2025). Asia is a region of aflatoxin concern after Africa, but it appears multiple Asian countries are taking steps to improve aflatoxin control and reduce the risks associated with aflatoxin exposure as they recorded the second highest number of studies (8). Europe has been very consistent in the control of aflatoxins and remains the region with the most established and organized aflatoxin control system globally. Continuous monitoring through different studies is paramount for regulatory decisions and effective control (Battilani et al. 2016).

**Figure 2:**
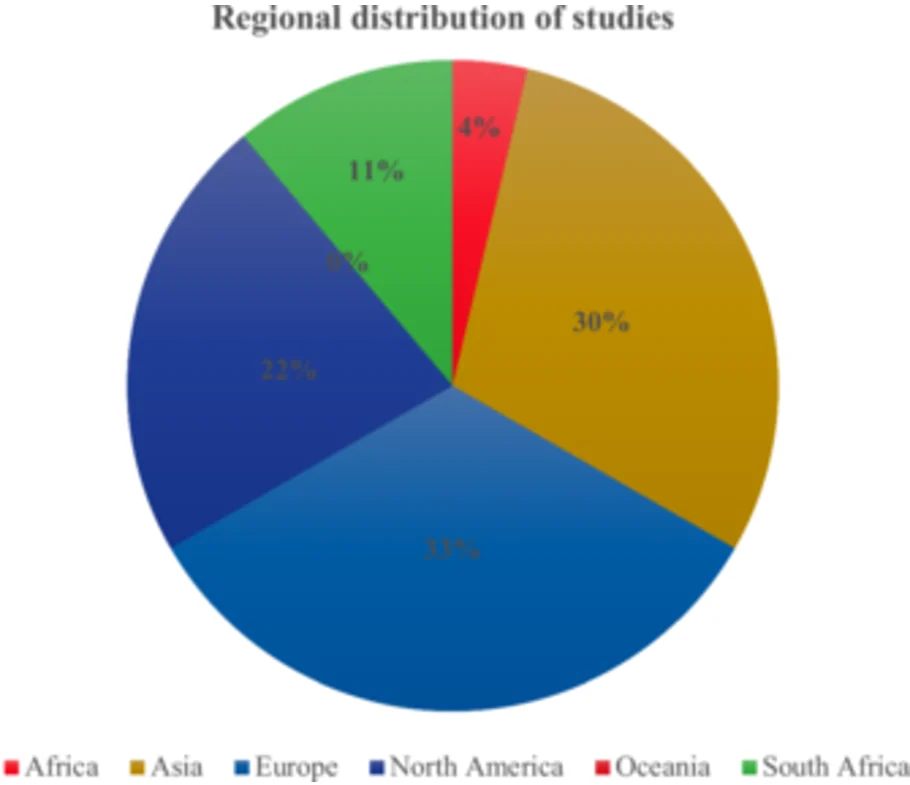
Regional percentage of articles on aflatoxin carryover from dairy cattle feed to milk retrieved publications (2004–2024). [Color figure can be viewed at wileyonlinelibrary.com]

### 3.2 Analytical Techniques

The screening results include a diverse range of studies conducted from 2004 to 2024, utilizing various methodologies to quantify aflatoxin levels in both feed and milk. Techniques like High‐Performance Liquid Chromatography (HPLC) and enzyme‐linked immunosorbent assays (ELISA) are frequently referenced, indicating progress in the accurate detection of aflatoxin concentrations amongst others. Across the studies retrieved from the bibliography search, differing methods were used, but almost 50% of the studies used HPLC, followed in frequency by ELISA (Figure 3).

**Figure 3:**
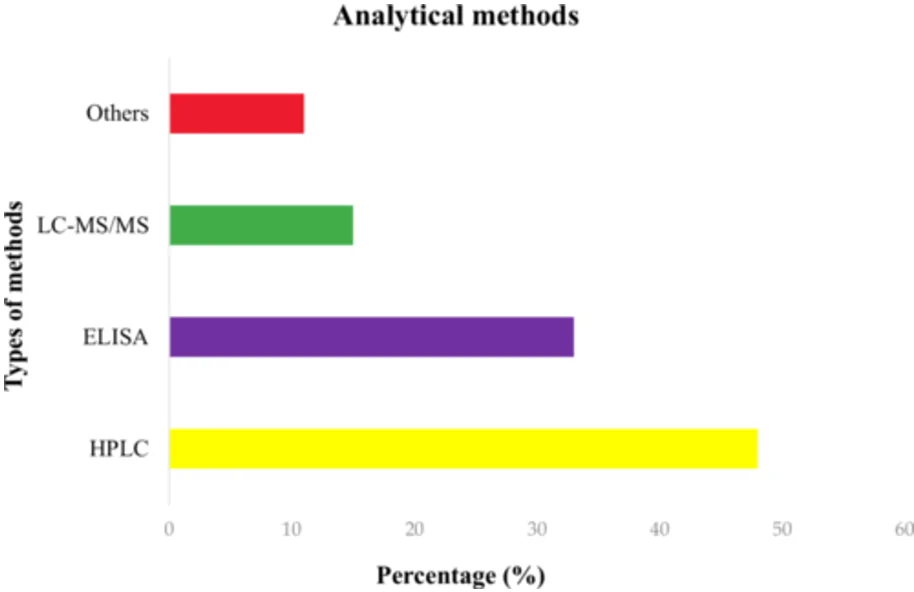
Percentage of articles using different methodologies for aflatoxin analysis in feed. [Color figure can be viewed at wileyonlinelibrary.com]

Developing analytical methods to detect aflatoxins in feeds is challenging when compared with milk due to their diverse chemical and physical structures, non‐homogenous mycotoxin distribution in feed, potential co‐occurrence of mycotoxins and the complex, variable composition of feed. Feed formulations differ based on raw materials, proportions, and specific nutritional needs for various animal species, ages, and seasons. Despite these challenges, it's crucial to create rapid, cost‐effective, and efficient detection methods to mitigate the risks of contaminated feed. Such methods should be accessible to small companies and control laboratories, leveraging affordable technologies. HPLC provides high precision, sensitivity, versatility, and the ability to analyse multiple components simultaneously, making it invaluable and reliable in feed and milk quality analysis. ELISA on the other hand is a powerful, versatile, and cost‐effective method for the analysis of feed and milk. It is especially advantageous when high throughput, rapid results, and specific detection of contaminants or nutrients are needed, making it a valuable tool in both quality control and safety monitoring. Other chromatographic methodologies were also used such as ultra‐performance liquid chromatography (UPLC) system (Borowsky et al. 2024) while the method applied in aflatoxin analysis in some studies was not clearly mentioned (Sulzberger et al. 2017). The development of new methodologies that facilitate the rapid, easy, and inexpensive analysis of mycotoxins in feed is essential for both research and feed control in small laboratories. Pate et al. (2018) used SNAP AFM_1_ test kit, a rapid test used to detect the presence of Aflatoxin M_1_ (AFM_1_) in milk. This detection technique identified aflatoxins in all samples, with most exceeding regulatory limits, suggesting high analytical sensitivity. While the method appeared technically successful, the unusually high detection rate underscores the need for cautious interpretation and further validation against established reference methods. Accordingly, the authors concluded that the method shows strong potential for improving aflatoxin surveillance and early detection but emphasised the need for additional studies comparing it with established reference methods to confirm its accuracy, reliability, and suitability for routine monitoring.

### 3.3 Feed and Milk Analyses

Numerous studies and datasets related to aflatoxins in feed and milk have been reported independently while very few reports have data that link aflatoxin contamination in feed to milk where carryover rate can be effectively determine. Out of the 27 articles selected for the study, 56% were designed as treatment studies evaluating the effectiveness and efficacy of diverse treatments (including chemical, physical and biological techniques; Table 2). When this was the case, results reported here are for the control treatments (Britzi et al. 2013; Diaz et al. 2004; Masoero et al. 2007; Walte et al. 2022). Ideally, non‐treatment studies should form the basis for discussion in terms of real‐life scenarios with regards to evaluating consumers’ exposure. In the manuscript, ‘treatment studies’ referred specifically to experiments where aflatoxin levels were artificially created by spiking feed with purified AFB_1_, in contrast to studies using naturally contaminated feed in which toxin levels arise from fungal growth under field or storage conditions. This distinction is important because animal responses to artificially fortified diets may underestimate real‐world toxicity. Numerous studies show that naturally contaminated feeds typically contain complex mixtures of mycotoxins and fungal metabolites many unquantified that exert additive or synergistic effects, leading to more severe biological responses than equivalent doses of purified AFB_1_ (Fusi et al. 2020; Gruber‐Dorninger et al. 2019; Streit et al. 2013). Other studies have demonstrated that mycotoxin mixtures amplify hepatic, immunological, and growth‐related effects (Alassane‐Kpembi et al. 2017; Grenier and Oswald 2011; Smith et al. 2016). Bryden (2012) and Fink‐Gremmels (2008) further note that these co‐contaminants can intensify toxicity beyond that observed in purified‐toxin challenges. For these reasons, distinguishing natural from artificially induced contamination enhances interpretation of treatment effects and strengthens the real‐world relevance of animal exposure outcomes. However, treatment studies further enrich this review toward the most efficient existing solution towards the aflatoxin‐contaminated feed.

**Table 2:** Aflatoxin levels in feed and milk and their carryover rates.

Treatment studies					Non‐treatment studies				
Author(s)	Year	AFB_1_ concentration (µg/kg)	AFM_1_ concentration (µg/kg)	Carryover rate (%)	Authors	Year	AFB_1_ concentration (µg/kg)	AFM_1_ concentration (µg/kg)	Carryover rate (%)
Kutz et al.	2009	112	1.00–1.98	1.42	Diaz et al.	2004	55	0.43–1.24	0.95–2.25
Polonelli et al.	2011	10.8–144	0.095–0.18	N/A	Masoero et al.	2007	98.0–98.3	1.27–2.66	1.29–2.70
Sumantri et al.	2012	3.4–350	0.002–0.069	0.033–0.207	Oliveira et al.	2008	1.4–26.4	0.010–0.645	0.71–2.44
Xiong et al.	2015	20‐40	17.0–17.6	0.057–0.59	Britzi et al.	2013	84.1–89.8	0.02–0.256	2.1–15.2
Gonçalves et al.	2017a	480	0.49–1.04	0.20–3.95	Rodríguez‐Blanco et al.	2019	0.05–6.45	0.009–1.36	0.6–6
Sulzberger et al.	2017	100	0.35–22.82	0.5–1.01	Costamagna et al.	2019	1.9–8.1	0.003–0.064	0.05–5.93
Jiang et al.	2018	16.0–28.5	0.40–0.75	0.93–1.65	Djekic et al.	2020	0.813–29.424	0.012–0.779	1.48–2.65
Xiong et al.	2018	20	0.13–7.38	0.11–1.38	Bervis et al.	2021	11.08–28.26	0.35–0.75	1.89–5.07
Pate et al.	2018	100	0.09–12.4	0.39–0.49	Walte et al.	2022	50	0.018–0.070	1.22–4.43
Guo et al.	2019	63	0.48–0.67	0.76–1.06	Hassan H. Al‐Rubaye	2023	5.37–6.53	0.006–0.008	0.28–0.44
Rodrigues et al.	2019	101.4–107.6	8.1–22.2	1.0–2.7	Jauković	2024	2.05–48.31	0.02–0.26	0.53–0.98
Intanoo et al.	2020	22.28	0.36–1.54	1.18–7.26	Borowsky et al.	2024	ND	0.03–0.32	NA
Cha et al.	2021	168	0.046–0.093	0.57–1.16					
Kemboi et al.	2023	326–598	0.689–2.737	0.05–0.35					
Costamagna et al.	2024	2.31	0.008–0.016	0.77–2.19					

Aflatoxin B_1_ contamination of feed for non‐treatment studies naturally ranged between 0.05 and 48.31 µg/kg, while set contamination for treatment was between 3.4 and 480 µg/kg. While the AFB_1_ concentrations of the feed in some treatments was fixed, others were programmed incrementally over a given period. It is worth noting that while some countries rarely regulate aflatoxins in feed, those that do typically choose to regulate either total aflatoxins or aflatoxin B_1_. Very few countries regulate both. EU member countries, for example focus exclusively on aflatoxin B_1_, while Asian countries regulate only total aflatoxins. Countries in other continents either regulate both aflatoxin B_1_ and total aflatoxins or just one of them. Since AFM_1_ is the hydroxylated product of AFB_1_, values reported in this study for feed contamination are those of AFB_1_ (Tables 1 and 2). From the articles reviewed, it is difficult to determine whether the reported AFB_1_ levels in feed should be considered ‘high,’ particularly in non‐treatment studies. This ambiguity arises because regulatory limits for AFB_1_ vary widely across countries and commodities, meaning that a concentration deemed acceptable in one jurisdiction may exceed the limit in another. For example, countries like Senegal and Algeria have very high regulatory limit of 300 µg/kg (FAO 2004), and Australia/New Zealand AFB_1_ regulatory limit in feed is as low as 5 µg/kg (Cressey 2009). The most common AFB_1_ regulatory limit in feed is 20 µg/kg which is applied in the European Union countries (FAO 2004). Furthermore, regulatory thresholds do not always align with biological or health‐based risk, as even relatively low concentrations especially under natural co‐contamination may still produce adverse effects in animals. Therefore, the interpretation of what constitutes ‘high’ contamination depends on both regulatory context and toxicological significance.

Aflatoxin M_1_ contamination of milk throughout all the studies under investigation ranged between 0.002 and 22.2 µg/kg. There are generally two common regulatory limits for aflatoxin M_1_ in milk (0.05 and 0.5 µg/kg). Most countries in Africa and Europe adopt the stricter 0.05 µg/kg limit for milk and milk products, whereas Asia, Australia, New Zealand, the United States, and countries in the Americas typically use the 0.5 µg/kg limit (FAO 2004). Regulatory frameworks are crucial for managing aflatoxin contamination and safeguarding consumer health, particularly for vulnerable populations like infants. In the non‐treatment studies, levels of AFM_1_ in milk reported in these studies are of safety concerns except for a few whose AFM_1_ levels were remarkably low regardless of the regulatory limit (0.05 or 0.5 µg/kg) (Costamagna et al. 2019; Al‐Rubaye and Atiyah 2023; Walte et al. 2022). On the other hand, levels of AFM_1_ in the treatment varied greatly and was influenced by the initial levels of AFB_1_ dosing as well as the efficiency of the treatment applied. While the sole purpose of treatment studies was to evaluate the efficacy of diverse treatment options, artificial contamination of feed with AFB_1_ of up to 598 µg/kg in some studies were reported (Kemboi et al. 2023). This is the reason why high levels of AFM_1_ were reported in milk from treatment studies.

The carryover rate reported in this review varied between 0.05% and 15.2% (including studies associated with experimental conditions involving artificially contaminated feed used for treatment or controlled exposure purposes). These statistics underscore the potential health hazards associated with aflatoxin exposure in dairy products, highlighting the urgent need for consistent monitoring of aflatoxin levels in feed to protect public health. No carryover values were reported in some studies (Borowsky et al. 2024; Polonelli et al. 2011). Most reports have reported carryover rates in the range of 1%–6% (Völkel et al. 2011; Nji et al. 2022). This is similar to what was observed in this review article except for the study by Britzi et al. (2013), with reported carryover rate slightly above 15% in mid‐lactation cow. Several critical factors affect the transfer of aflatoxins from feed to milk, including but not limited to storage conditions of feed, (particularly temperature and moisture), lactation stage, AFB_1_ contamination doses, duration of exposure, health status, species of cow, and age (Nji et al. 2022). Recognizing these factors is vital for developing effective interventions to reduce aflatoxin levels in dairy products. The findings of Borowsky et al. (2024) where AFB_1_ was undetected in feed but measurable AFM_1_ was present in milk illustrate the well‐documented challenge of obtaining representative feed samples (Whitaker 2006). Mycotoxins are known to occur heterogeneously within bulk material, and inadequate sampling can result in false negatives, thereby undermining the reliability of feed testing and complicating data interpretation (Whitaker 2006). This example underscores a key limitation in mycotoxin surveillance and highlights the importance of robust sampling strategies. Better still, one might be tempted to attribute no AFB_1_ detection in feed samples to errors in the clean‐up procedure or matrix effect in the analytical method (ultra‐performance liquid chromatography (UPLC) system). This chromatographic technique has previously been successfully applied in the quantification of feed and milk samples by (EC 2023; Franco et al. 2019) respectively. Hence, aside sampling issues, the calibration process and matrix effects might not have been sufficiently addressed.

### 3.4 Factors Influencing Aflatoxin Carryover to Milk

Carryover of AFB_1_ from feed into AFM_1_ in milk is influenced by several factors which could be categorised as nutritional, physiological, and environmental (Rodriguez‐Blanco et al. 2019). While over 60% of the extracted studies reviewed these factors, Al‐Rubaye and Atiyah (2023) on a comparative study extensively showed and reported how aflatoxin carryover from feed to raw milk differ in dairy animal species. Aflatoxin carryover to milk is a multifactorial process shaped by dietary composition and contamination, animal physiology, and management practices. While multiple variables contribute, milk yield, feed AFB_1_ concentration, rumen detoxification capacity, and mammary gland integrity are among the strongest determinants. Effective control requires integrated dietary, physiological, and management interventions targeting each of these domains.

#### 3.4.1 Dietary Factors

Dietary characteristics are reported among the strongest determinants of aflatoxin carryover because they directly influence the level of AFB_1_ exposure, the rate of ingestion, and the ability of the rumen and liver to detoxify the toxin. Carryover, as will be expected, largely reflects the concentration of AFB_1_ in the feed. Feeds such as cottonseed, maize, soybean expeller, and complex concentrates are consistently linked to higher AFB_1_ levels (Costamagna et al. 2019). In many studies, these ingredients contribute more than half of total AFB_1_ intake. Stored feeds are especially prone to contamination, particularly under hot and humid conditions. Conditions during harvest, drying, and storage significantly affect fungal growth and aflatoxin production in feed. Poorly stored feeds, especially during cold or wet seasons, accumulate more AFB_1_, increasing exposure and elevating AFM_1_ excretion in milk. Furthermore, the rate of feed intake influences how quickly AFB_1_ enters systemic circulation. Animals consuming large amounts of contaminated concentrates common in high‐yielding cows absorb more AFB_1_, increasing AFM_1_ transfer (Ferrari et al. 2023; Soriano del Castillo 2007; Battacone et al. 2009).

Feeding systems relying heavily on stored concentrates or poorly mixed total mixed ration (TMR) increase exposure and reduce detoxification efficiency. In contrast, grazing‐based systems lower risk by providing fresh forage with minimal AFB_1_ contamination. The rumen has natural detoxifying capacity, but this depends on pH stability, retention time of feed particles and microbial population balance. Improper TMR particle size causes selective feeding on fine particles that rapidly exit the rumen, shortening detoxification time (Costamagna et al. 2019). Grain‐rich diets fed to high‐producing cows lower rumen pH, promoting subacute ruminal acidosis and reducing microbial degradation of AFB_1_.

#### 3.4.2 Physiological Factors

Physiological variables determine how animals absorb, metabolize, and excrete aflatoxins, influencing the proportion of AFB_1_ that appears in milk as AFM_1_. These include factors such as milk production rate, lactation stage, animal species, breed and age, liver detoxification capacity, metabolic status among others (Masoero et al. 2007; Costamagna et al. 2019). Milk yield is the most influential factor. Low‐yield cows typically exhibit 1%–2% carryover, while high‐yield cows may reach 5%–6% (Ferrari et al. 2023). Increased metabolic throughput, greater feed intake, and altered plasma–milk barrier permeability in high‐yielding animals contribute to higher transfer rates (Costamagna et al. 2019). Cows in early lactation (2–4 weeks postpartum) show the highest carryover due to peak milk yield, increased metabolic load, and elevated feed intake. Carryover declines as lactation progresses and milk production decreases.

Al‐Rubaye and Atiyah (2023), on a comparative study of AFM1 carryover from feed to raw milk in different dairy animal species, concluded that species have intrinsic differences in susceptibility: sheep and goats tend to transfer AFB_1_ more efficiently than cattle or buffaloes. Hence, breed‐specific metabolic capacities and age‐related differences in rumen development or liver enzyme activity also modify carryover rates (Al‐Rubaye and Atiyah 2023). Furthermore, liver detoxification capacity also play an important role. AFB_1_ metabolism depends on cytochrome P450 enzymes and glutathione‐S‐transferase. Animals with more robust detoxification systems convert less AFB_1_ into AFM_1_. Conversely, impaired hepatic function elevates carryover. High metabolic demands in early lactation, negative energy balance, or stress can alter detoxification pathways and increase AFM_1_ secretion. Individual differences in rumen degradation of AFB_1_, including conversion to other metabolites such as aflatoxicol, influence AFM_1_ formation. Integrity of blood–milk barrier and mammary epithelial cells affect carryover rates. AFM_1_ transfer depends on the permeability of mammary epithelial membranes (Britzi et al. 2013; Masoero et al. 2007). When this integrity is compromised (e.g., inflammation), passive transfer into milk increases. Udder health and alveolar cell membrane condition are reported to influence aflatoxins carryover. Mastitis elevates somatic cell counts and disrupts membrane permeability, potentially increasing AFM_1_ carryover (Masoero et al. 2007). Although evidence varies, inflammation generally weakens the mammary barrier.

#### 3.4.3 Management and Environmental Factors

Management practices and environmental conditions influence both the quality of feed and the physiological state of animals, indirectly affecting aflatoxin carryover. Cold and dry seasons, or periods of limited grazing, force reliance on stored or mixed feeds that are more prone to AFB_1_ contamination (Jaukovic et al. 2024; Sulzberger et al. 2017). Conversely, grazing seasons reduce exposure due to the consumption of fresh forage (Jaukovic et al. 2024). Regions with high humidity, fluctuating temperatures, or inadequate storage infrastructure experience greater aflatoxin contamination, leading to higher AFM_1_ levels in milk. Illness, stress, and compromised immunity reduce metabolic efficiency and digestive function, weakening detoxification of AFB_1_ and increasing AFM_1_ excretion (Goncalves et al. 2017b). Poor mixing, excessive long fibers, or inconsistent TMR particle size encourage feed sorting, alter rumen pH stability, and reduce rumen detoxification capacity, thereby elevating carryover (Costamagna et al. 2019). High‐producing dairy systems, which rely on intensive concentrate feeding, increase aflatoxin exposure and physiological susceptibility to higher carryover rates. Updated risk assessments must consider modern feeding and management contexts.

### 3.5 Aflatoxin Mitigation Strategies in Feed and Milk and Regulatory Standards

AFs pose significant risks to both livestock and humans. AFB_1_ is classified as a Group 1 carcinogen by the International Agency for Research on Cancer (IARC 2012) due to its strong association with hepatocellular carcinoma, mutagenicity, teratogenicity, and immunosuppressive effects (Alameri et al. 2023). AFM_1_, the primary milk metabolite of AFB_1_, is also a Group 1 carcinogen and represents a critical food safety concern, particularly for infants and children (IARC 2012). In livestock, aflatoxicosis manifests acutely as reduced feed intake, lethargy, reproductive disorders, and weight loss (Popescu et al. 2022). Chronic exposure in dairy cows reduces milk yield, milk fat and protein content, and may impair liver function, immune status, and vaccine responsiveness (Queiroz et al. 2012; R. Rodrigues et al. 2019). Up to 6% of dietary AFB_1_ can be transferred into milk as AFM_1_, further increasing public health risks (Zentai et al. 2023). Toxicity mechanisms involve reactive oxygen species (ROS), DNA/RNA adduct formation, and induction of inflammatory pathways, including upregulation of NFKB1 and GPX1 (Dai et al. 2024).

Given the severe health and economic impacts of aflatoxins (AFs) on both humans and animals, the establishment and enforcement of regulatory standards are critical for limiting exposure. Globally, over 60 countries regulate AFM_1_ in milk, and more than 100 countries set limits for AFB_1_ in feed. Regulatory thresholds vary across regions:

- *European Union (EU)*: AFB_1_ ≤ 5 µg/kg in feed; AFM_1_ ≤ 0.05 µg/kg in milk; ≤ 0.025 µg/kg in infant milk (Bürgin 2021).
- *United States (FDA)*: AFB_1_ ≤ 20 µg/kg in feed; AFM_1_ ≤ 0.5 µg/kg in milk (Fleming 2000).
- *China*: AFB_1_ ≤ 10 µg/kg in feed; AFM_1_ ≤ 0.5 µg/kg in milk (Liu et al. 2011).
- Other regions, including Serbia, Thailand, and MERCOSUR, maintain comparable limits with minor variations (Bürgin 2021; de Oliveira and Corassin 2014).

Despite these standards, AFM_1_ contamination remains prevalent, largely due to inconsistent monitoring capacity, particularly in developing countries (Mutegi et al. 2018). Most international regulations adopt either 0.05 or 0.5 µg/kg as the maximum limit for AFM_1_ in milk, depending on country or regional policy. Compliance requires robust surveillance systems, integrating rapid screening assays with confirmatory chromatographic techniques; however, analytical and logistical capacity gaps continue to impede effective implementation in low‐resource settings (Mutegi et al. 2018).

Sustainable mitigation of aflatoxins in feed and milk necessitates integrated, multi‐level interventions. Preventive measures such as improved field practices, controlled drying, proper storage, and feed hygiene remain foundational. These must be complemented by detoxification technologies, use of mycotoxin binders, and routine milk quality monitoring throughout the dairy value chain. Emerging innovations including digital traceability systems, farmer training, participatory surveillance, and biotechnology‐based solutions such as gene‐edited crops for enhanced resistance and microbial enzyme detoxification offer additional opportunities to strengthen control (Alberts et al. 2019; Frazzoli et al. 2016). Overall, the persistence, stability, and toxicity of aflatoxins underscore the need for holistic, regulation‐driven, and farmer‐inclusive strategies. Coordinated action across production, processing, regulation, and education is essential to safeguard public health and ensure the long‐term sustainability of the dairy sector.

The regulatory framework for aflatoxins in dairy feed and milk varies considerably across countries, resulting in uneven consumer protection; nations with more stringent feed limits generally report lower aflatoxin levels in milk (Xiong et al. 2015). Mitigating aflatoxin exposure in dairy systems therefore requires integrated, multi‐level interventions aimed at preventing contamination, reducing gastrointestinal bioavailability, and detoxifying contaminated feed. Prevention remains the most effective strategy, beginning in the field through good agricultural practices (GAPs) timely planting, crop rotation, resistant varieties, irrigation management, and biocontrol using atoxigenic *Aspergillus flavus* strains which can reduce AFB_1_ by up to 80% (Atehnkeng et al. 2016; Bandyopadhyay et al. 2019; Ouadhene 2023). Post‐harvest practices such as rapid drying (< 13% moisture), proper cleaning, controlled temperature–humidity storage, and hermetic systems further limit fungal growth and toxin accumulation (Fusseini 2015; Hell and Mutegi 2011; Kabak et al. 2006). Because AFM_1_ carryover into milk typically ranges between 1% and 6% of dietary AFB_1_ intake (Fink‐Gremmels 2008), reduction at the feed level is crucial. Among mitigation options, adsorbents such as bentonite, kaolin, montmorillonite, hydrated sodium calcium aluminosilicate, glucomannan, activated carbon, and yeast cell wall derivatives effectively bind AFB_1_ in the gastrointestinal tract and lower AFM_1_ secretion in milk (Kabak et al. 2006; Rodrigues et al. 2019; Walte et al. 2022). However, efficacy varies with dose, contamination level, and adsorbent type and source, and excessive inclusion may impair nutrient utilization or reduce milk yield (H. Jiang et al. 2018; S. Sulzberger et al. 2017). Yeast‐based products such as *Saccharomyces cerevisiae* fermentation products enhance gut health, modulate inflammatory responses, and improve performance under AFB_1_ exposure, while combinations of yeast and clay may enhance sequestration efficiency (Jensen et al. 2015; Ogunade et al. 2016; Poppy et al. 2012). Biological detoxification using microbial enzymes (e.g., laccases), lactic acid bacteria, and degrading microbes offers a promising, residue‐free approach (Guo et al. 2019; Intanoo et al. 2020; Smaoui et al. 2023). Physical treatments including thermal processing, irradiation, sorting, and extrusion provide partial reduction but are often impractical or costly at scale (Guo et al. 2019; Sipos et al. 2021). Chemical detoxification methods such as ammoniation and ozonation are effective but limited by concerns over residues and regulatory acceptance (Kolosova and Stroka 2011).

These studies conclusively indicate that AFB1 concentration reduction in dairy cow diets is feasible, though the efficacy varies with type and source of adsorbent, AFB1 contamination levels, dose of treatment, and consistency of additive intake (Table 3).

**Table 3:** Aflatoxin reduction in milk observed in research trials with various adsorbents.

#	Author	Year	Feed treatment technique	Aflatoxin reduction efficacy from feed to milk (%)	Baseline AFB_1_ in feed (µg/kg)	Baseline AFM_1_ in milk (µg/kg)
1	Kutz et al.	2009	Solis	45	100–112	0.57
			NovasilPlus	48		
			MTB‐100	4		
2	Polonelli et al.	2011	Anaflatoxin B1 conjugated to keyhole limpet hemocyanin as a vaccine	37	80–100	0.30–0.40
3	Sumantri et al.	2012	Bentonite	No significant effect	30.8	150–200 ng/kg
4	Xiong et al.	2015	Solis Mos	22	63–100	0.2–0.5
5	Gonçalves et al.	2017a	Saccharomyces cerevisiae	89	75	0.2–0.4
6	Sulzberger et al.	2017	Clay	37	102	0.3–0.5
7	Jiang et al.	2018	Clay	39	100	0.2–0.4
			Clay + Saccharomyces cerevisiae	43		
8	Xiong et al.	2018	Solis Mos	36	60–100	0.3–0.6
9	Pate et al.	2018	Aluminosilicate clay	20	102	0.3
10	Guo et al.	2019	*Bacillus subtilis* ANSB060	72	50–80	0.2–0.4
11	Rodrigues et al.	2019	Toxy‐Nil	63	50–100	0.2–0.4
			Unike Plus	52		
12	Intanoo et al.	2020	Kluyveromyces marxianus,	72	50–80	0.2–0.4
			Pichia kudriavzevii			
13	Cha et al.	2021	Adsorbent 1 = (Montmorillonite + diatomite)	51	60–100	0.3–0.5
			Adsorbent = (Montmorillonite + diatomite + yeast extracts + sodium alginate)	46		
14	Kemboi et al.	2023	Bentonite (Crosses and Borans)	30	19–47	128–358 ng/kg
				43		
15	Costamagna et al.	2024	AntitoxCooPil + 60% zeolite + 40% cell wall	65	80–100	0.3–0.6

Once AFM_1_ enters milk, its high stability renders conventional processing largely ineffective as a means to reduce aflatoxin contamination; pasteurization and sterilization cause minimal reduction, while cheese‐making reduces levels through partitioning but does not eliminate residues (Flores‐Flores et al. 2015; Prandini et al. 2009). Emerging technologies including cold plasma, UV‐C irradiation, and enzymatic degradation show potential for safer milk decontamination (Misra et al. 2019). Overall, effective AF mitigation in dairy systems relies on coordinated preventive practices, strategic use of binders and biological agents, and robust monitoring to limit carryover into milk and protect public health (Van der Fels‐Klerx et al. 2019).

## 4 Conclusion

Dairy cattle contribute the vast majority of global milk production due to their high adaptability, efficient feed conversion, and superior milk yields. However, contamination of feed with mycotoxins particularly aflatoxins, poses a significant threat to both animal productivity and milk safety by compromising feed quality and reducing milk output. This systematic review highlights the transfer of aflatoxins from contaminated animal feed into dairy milk, demonstrating that aflatoxin metabolites particularly AFM_1_ can enter the human food chain. In several cases, the concentrations detected in milk reached levels that raise significant public health concerns. Vulnerable populations, especially infants and young children, are at heightened risk, as chronic exposure has been linked to immunosuppressive and carcinogenic effects. The wide variability in AFM_1_ levels across studies, influenced by feed quality, storage practices, and seasonal changes, calls for more robust monitoring and risk management strategies. Although global regulatory limits exist, inconsistencies between countries and a lack of harmonized enforcement mechanisms hinder effective consumer protection. These gaps are especially pronounced in sub‐Saharan Africa (SSA), where limited surveillance infrastructure and inadequate food consumption data further restrict meaningful risk assessments. As such, urgent action is required to strengthen food safety monitoring and harmonize regulatory frameworks across regions. In response, initiatives like the EU‐AU funded HORIZON UP‐RISE project (HORIZON‐CL6‐2023‐FARM2FORK‐01101136649) are addressing these critical gaps. The project specifically investigates aflatoxin carry‐over from feed into milk in South Africa, aiming to develop integrated food safety solutions tailored to local agricultural systems. Insights from this ongoing work will provide essential data on how climate change exacerbates mycotoxin contamination, thereby informing policies and practices that can reduce aflatoxin exposure in the dairy value chain. Ultimately, tackling aflatoxin contamination in the dairy sector demands coordinated efforts among researchers, policymakers, producers, and consumers. By prioritizing surveillance, education, and regulatory alignment, it is possible to enhance milk safety, protect public health, and build more resilient food systems in Africa and beyond.

## Conflicts of Interest

The authors declare no conflicts of interest.

## Animal Welfare Statement

The authors confirm that the ethical policies of the journal, as noted on the journal's author guidelines page, have been adhered to. No ethical approval was required as this is a review article with no original research data.

## Supporting information


Supporting File 1

Supporting File 2

## Data Availability

The data that support the findings of this study are available on request from the corresponding author. The data are not publicly available due to privacy or ethical restrictions.
