# Supplementary material for: Carryover of Aflatoxin From Dairy Feed to Milk: A Systematic Review (1 January 2004–31 December 2024)
Source: J Anim Physiol Anim Nutr (Berl). 2026 Jul 19;110(6):821–36. doi: 10.1111/jpn.70092 (PMC13768934; doi:10.1111/jpn.70092)
Supplement: Supplementary file 1 [file JPN-110-821-s001.docx]

JBI Critical Appraisal Checklist for
systematic reviews and research syntheses

Reviewer	___Patrick____Njobeh______ Date_______7__June______________________

Author_Richard T. _Fagbohun_____ Year__2025_______ Record Number____27_____

|  | Yes | No | Unclear | Not applicable |
| --- | --- | --- | --- | --- |
| 1. Is the review question clearly and explicitly stated? | □  | □ | □ | □ |
| 2. Were the inclusion criteria appropriate for the review question? | □  | □ | □ | □ |
| 3. Was the search strategy appropriate? | □  | □ | □ | □ |
| 4. Were the sources and resources used to search for studies adequate? | □  | □ | □ | □ |
| 5. Were the criteria for appraising studies appropriate? | □  | □ | □ | □ |
| 6. Was critical appraisal conducted by two or more reviewers independently? | □  | □ | □ | □ |
| 7. Were there methods to minimize errors in data extraction? | □  | □ | □ | □ |
| 8. Were the methods used to combine studies appropriate? | □  | □ | □ | □ |
| 9. Was the likelihood of publication bias assessed? | □  | □ | □ | □ |
| 10. Were recommendations for policy and/or practice supported by the reported data? | □  | □ | □ | □ |
| 11. Were the specific directives for new research appropriate? | □  | □ | □ | □ |

Overall appraisal:	Include	□	Exclude	□	Seek further info □

Comments (Including reason for exclusion)

________This systematic review is commendable for its thoroughness and adherence to the JBI Critical ________________________________________________________________________________________________________________________________________________________________________________________________________________________________________________________________________________________
