# Supplementary material for: Carryover of Aflatoxin From Dairy Feed to Milk: A Systematic Review (1 January 2004–31 December 2024)
Source: J Anim Physiol Anim Nutr (Berl). 2026 Jul 19;110(6):821–36. doi: 10.1111/jpn.70092 (PMC13768934; doi:10.1111/jpn.70092)
Supplement: Supplementary file 2 [file JPN-110-821-s002.docx]

# PRISMA 2020 Flow Diagram

## Identification

Records identified from databases: 228

Records identified from other sources (manual searches, references): 57

Total: 285

## Screening

Records after duplicates removed: 171

Records screened (titles/abstracts): 171

Records excluded (irrelevant, not Aflatoxin carries over, etc.): 144

## Eligibility

## Full-text articles assessed: 103

**Full-text articles excluded**: 76 Human study =1, no data on Aflatoxin M= 8, Wrong comparators n=24, Wrong study designs n = 2, language barrier n=1, Data on Aflatoxin in feeds not included n = 14, Not in the design scope of this study n = 10)

## Included

Studies included in qualitative synthesis: 27

Studies included in quantitative synthesis (systematic review tables): 27
